# Genome-Wide Development and Validation of KASP-Based SNP Markers in *Neophocaena asiaeorientalis asiaeorientalis*

**DOI:** 10.3390/ani16030475

**Published:** 2026-02-03

**Authors:** Denghua Yin, Han Zhang, Mengting Tang, Jianglong Que, Danqing Lin, Congping Ying, Jialu Zhang, Jinxiang Yu, Kai Liu

**Affiliations:** 1 Wuxi Fisheries College, Nanjing Agricultural University, Wuxi 214081, China; yindenghua@ffrc.cn (D.Y.); lindq@ffrc.cn (D.L.); yingcongping@ffrc.cn (C.Y.); zhangjialu@ffrc.cn (J.Z.); 2 Key Laboratory of Freshwater Fisheries and Germplasm Resources Utilization, Ministry of Agriculture and Rural Affairs, Freshwater Fisheries Research Center, Chinese Academy of Fishery Sciences, Wuxi 214081, China; 3 National Demonstration Center for Experimental Fisheries Science Education, Shanghai Ocean University, Shanghai 201306, China; hanzhang0221@163.com (H.Z.); m230150512@st.shou.edu.cn (M.T.); 4 Aquatic Conservation and Rescue Center of Jiangxi Province, Nanchang 330096, China; que_jianglong@sina.com

**Keywords:** Yangtze finless porpoise, SNP, KASP, genetic diversity, molecular marker

## Abstract

The Yangtze finless porpoise (*Neophocaena asiaeorientalis asiaeorientalis*) is the only extant freshwater cetacean species inhabiting the Yangtze River in China. Facing significant threats from habitat degradation and human activities, this critically endangered species requires urgent conservation efforts. A thorough understanding of its genetic diversity is fundamental for informing effective conservation strategies. To address the need for stable and reliable molecular markers, this study aimed to develop and validate a set of highly polymorphic single nucleotide polymorphism (SNP) markers. Candidate SNPs were initially identified based on chromosome-level genome and whole-genome resequencing data, yielding a total of 1070 candidate loci. Following the principle of even distribution across chromosomes, 50 SNPs were randomly selected for Sanger sequencing validation, from which 35 polymorphic SNPs were preliminarily confirmed. These 35 SNPs were subsequently genotyped using the Kompetitive allele-specific PCR (KASP) assay, which successfully validated 19 highly polymorphic markers. Genetic diversity analysis using these SNPs successfully captured moderate-to-high polymorphism in the Poyang Lake population, demonstrating the capability of these markers in detecting genetic variation. The SNP markers developed in this study show promising applicability for genetic diversity assessment in the Yangtze finless porpoise. Further validation across broader geographic and demographic samples will be essential to confirm their potential efficiency for detecting spatial heterogeneity and informing region-wide management strategies.

## Simple Summary

The critically endangered Yangtze finless porpoise urgently requires effective conservation strategies, for which understanding its genetic diversity is essential. This study developed a suite of reliable molecular markers to support such efforts. By integrating chromosome-level genome data with whole-genome resequencing, candidate genetic variants were identified and subsequently validated using high-throughput Kompetitive Allele-Specific PCR (KASP) technology. A total of 19 highly polymorphic single nucleotide polymorphism (SNP) markers were successfully established. Population analysis employing these markers revealed higher genetic diversity in the Poyang Lake population, demonstrating their potential efficiency in detecting genetic variation. These newly developed SNP markers provide a practical and robust tool for future population genetics research and informed conservation management of the Yangtze finless porpoise.

## 1. Introduction

The Yangtze finless porpoise (*Neophocaena asiaeorientalis asiaeorientalis*), belonging to the suborder Odontoceti and family Phocoenidae, represents the only exclusively freshwater cetacean inhabiting the middle-lower Yangtze River mainstream and its adjacent lake systems including Poyang and Dongting [1,2,3]. As a critical indicator species for ecosystem integrity, its population status directly reflects the ecological health of the entire river basin [4]. In recent decades, intensive anthropogenic activities and rapid economic development have triggered severe environmental degradation, leading to catastrophic declines in aquatic biodiversity [5]. By 2022, the population of this endemic species had plummeted to approximately 1249 individuals, pushing it to the brink of extinction. Consequently, the Species Survival Commission of the International Union for Conservation of Nature (IUCN/SSC) classified it as Critically Endangered (CR) in 2013 [6], followed by its elevation to a National First-Class Protected Animal in China in 2021. While current conservation strategies predominantly address external threats like human disturbance [7,8,9,10] and climate change [11,12], the species’ long-term survival equally depends on its intrinsic genetic constitution and evolutionary potential. A thorough understanding of the population genetics of the Yangtze finless porpoise, including its genomic diversity and population structure, is therefore essential for guiding the implementation of effective, science-based conservation strategies.

Genetic markers serve as fundamental tools in population genetic research. The evolution of molecular techniques has advanced genetic markers from second-generation systems, including microsatellites (SSR) [13,14] and amplified fragment length polymorphisms (AFLP) [15], to third-generation DNA markers represented by single-nucleotide polymorphisms (SNPs) [16,17,18,19]. SNPs offer unprecedented advantages through their genome-wide distribution, abundance, Mendelian inheritance patterns, precise genotyping accuracy, and compatibility with high-throughput automation, positioning them as ideal markers for conservation genomics [20]. Current genetic research on Yangtze finless porpoises remains constrained by reliance on microsatellite markers [21,22], which suffer from technical limitations including laborious development, genotyping inaccuracies, and low throughput, thereby impeding detailed population structure analysis. Although preliminary SNP development in *Neophocaena* [23,24] using fragment length differential allele-specific PCR (FLDAS-PCR) and comparative anchor tagged sequencing (CATS) demonstrated feasibility, these conventional approaches are hampered by procedural complexity, excessive costs, and suboptimal efficiency, rendering them inadequate for systematic genetic monitoring.

Recent breakthroughs in genome sequencing technologies have revolutionized SNP discovery by enabling systematic screening at chromosomal resolution [25]. Leveraging chromosome-level reference genomes complemented by population resequencing data, researchers can now identify genome-wide candidate SNPs with comprehensive coverage, overcoming the fundamental constraints of traditional methods in both locus density and genomic distribution. The transition from SNP discovery to practical application necessitates robust genotyping methodologies [26]. The Kompetitive allele-specific PCR (KASP) has emerged as a premier genotyping platform, combining fluorescence-based detection with unparalleled accuracy, cost-effectiveness, and operational simplicity, making it particularly suitable for non-model organisms [27,28]. The integration of genome-wide SNP discovery with KASP genotyping establishes a seamless pipeline from initial marker identification to experimental validation, successfully implemented in conservation genomic studies of endangered species including giant pandas [29]. This integrated approach proves particularly valuable for critically endangered species like the Yangtze finless porpoise, maximizing genetic information recovery from limited sample availability.

To establish an efficient and reliable SNP-based genetic marker system for Yangtze finless porpoises, this study integrated chromosome-level genomic resources with whole-genome resequencing data to screen evenly distributed, high-integrity candidate SNPs across the genome. These candidates underwent high-throughput genotyping and polymorphism validation through KASP technology. Our objectives were to develop a panel of highly polymorphic and technically robust SNP markers, evaluate their practical utility through genetic diversity assessment of the Poyang Lake population, and ultimately provide powerful technical support for conservation genetics, germplasm resource protection, and evidence-based genetic management strategies for this critically endangered species.

## 2. Materials and Methods

### 2.1. Ethical Approval

All experimental procedures involving Yangtze finless porpoises were conducted in accordance with China’s animal protection laws and the Regulations on the Protection of Aquatic Wildlife (promulgated 1993, revised 2013), following approval from the relevant regulatory authorities. All physical examination procedures, including animal chasing, handling, and blood collection, were approved under the official documents (Gan nong Zi [2022] No. 52 and [2022] No. 10) issued by the Department of Agriculture and Rural Affairs of Jiangxi Province. This study was conducted and reported in full adherence to the ARRIVE guidelines (https://arriveguidelines.org) (accessed on 19 August 2024).

### 2.2. Experimental Materials

**Figure 1:**
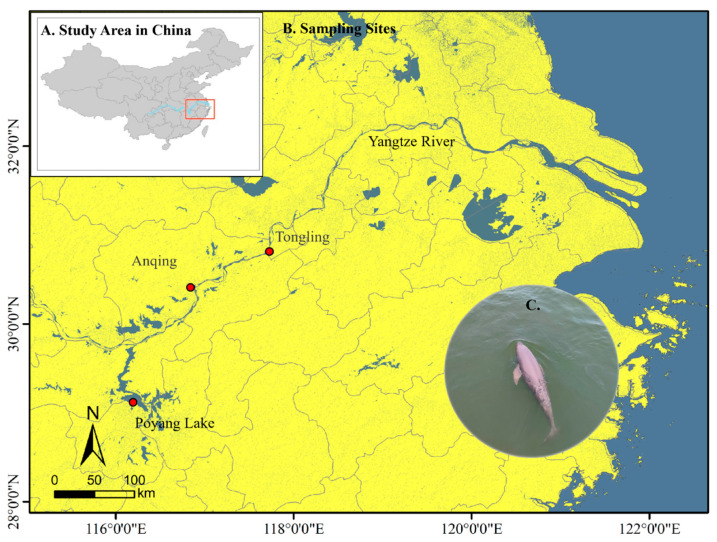
Sampling locations and study area of the Yangtze finless porpoise. (**A**) Map of China indicating the study region in the Yangtze River basin. (**B**) Detailed distribution of sampling sites within the study area. (**C**) Representative image of the Yangtze finless porpoise (*Neophocaena asiaeorientalis asiaeorientalis*).

For Sanger sequencing validation, two individuals were sampled from the Poyang Lake population. Blood samples for KASP genotyping (*n* = 28) were collected from ex situ populations during routine health examinations conducted at Tongling (*n* = 4, 2021) and Anqing Xijiang (*n* = 4, 2023), with both sites located along the Yangtze River. Additional samples were obtained from the Poyang Lake population during emergency rescues prompted by extreme drought conditions (*n* = 20, 2022–2023). A total of 30 blood samples from the Poyang Lake population, including the 20 previously used for KASP genotyping, were employed to validate SNP (Figure 1). All blood samples were collected from the tail vein using disposable sterile syringes, immediately transferred to EDTA-K2 anticoagulant vacuum tubes, and transported to the laboratory at −20 °C. Upon arrival, samples were stored long-term at −80 °C in ultra-low temperature freezers.

### 2.3. Screening of Polymorphic SNP Loci

Polymorphic SNP loci were identified using a chromosome-level genome assembly of the Yangtze finless porpoise (PRJNA915046) [30] and whole-genome resequencing data from 12 individuals (PRJNA1304169) [31]. The analytical procedure was conducted as follows (Supplementary Figure S1):

The sequence alignment and processing step began with aligning quality-controlled resequencing reads from the 12 samples to the reference genome using BWA-MEM (v0.7.19) [32] to generate SAM files. These files were converted to compressed BAM format, sorted by genomic coordinates, and indexed (.bai files) using SAMtools (v1.9) [33] to enable efficient data access. The resulting BAM files were then filtered to retain only high-quality alignments with a mapping quality (MAPQ) score greater than 30 using SAMtools, and subsequently re-indexed for downstream analysis.

For variant calling, initial variant discovery was performed for each sample using the HaplotypeCaller module in GATK (v4.6.2.0) [34], which produced genomic VCF (GVCF) files. Individual GVCFs were consolidated using GATK’s CombineGVCFs (v4.1.4.1), followed by joint genotyping of all samples via GenotypeGVCFs (v4.1.6.0) to generate a raw VCF file. SNPs were extracted from this file using GATK’s SelectVariants and subjected to hard filtering with the following criteria: --filter-expression “QD ≥ 2.0 && QUAL ≥ 30.0 && FS ≤ 60.0 && MQ ≥ 40.0 && MQRankSum ≥ −12.5 && ReadPosRankSum ≥ −8.0”. Only biallelic SNPs located in genomic intervals with no other variants within 100 bp upstream or downstream were retained. Subsequently, high-confidence SNPs with quality scores above 1000 were tagged using GATK’s VariantFiltration with the parameters: --filter-name “FIRSTOK” --filter-expression “QD ≥ 2.0 && QUAL ≥ 1000.0 && FS ≤ 60.0 && MQ ≥ 40.0 && MQRankSum ≥ −12.5 && ReadPosRankSum ≥ −8.0”. This step ensured the retention of high-quality SNP loci for downstream analyses.

**Figure 2:**
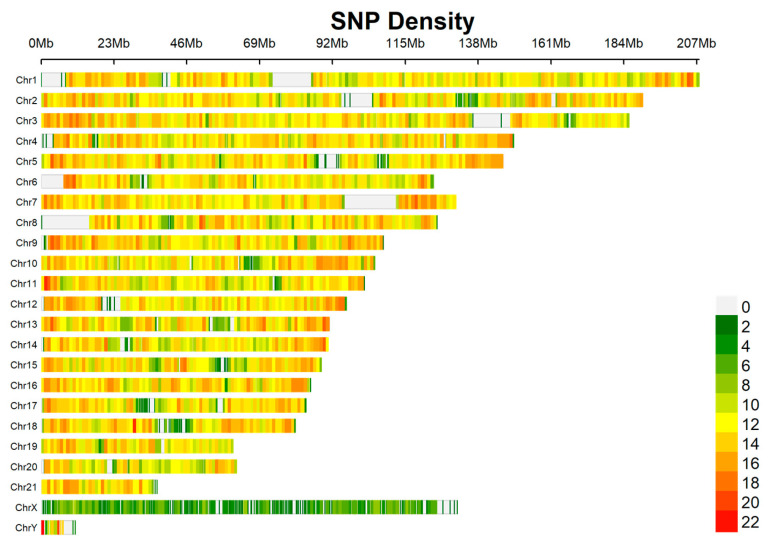
Chromosomal distribution density of the 29,177 candidate SNPs initially identified in the Yangtze finless porpoise genome.

Polymorphic SNP screening was conducted by further refining the initial SNP dataset using VCFtools (0.1.16) [35] to select high-quality, polymorphic loci suitable for subsequent Sanger sequencing validation. Filtering parameters included the following: --maf 0.05, --max-missing 1, --hwe 0.001. From this subset, we retained only biallelic SNPs with a polymorphism information content (PIC) value between 0.2 and 0.5, located in genomic regions where no other SNPs were present within 100 bp upstream or downstream. The final dataset comprised 29,177 candidate SNPs (Figure 2). To ensure broad genomic representation for downstream applications, a subset of 1070 SNPs evenly distributed across all chromosomes was randomly selected for further analysis (Supplementary Figure S2).

### 2.4. Sanger Sequencing Validation

To eliminate false-positive calls, a subset of 50 putative SNPs was randomly selected from the filtered polymorphic SNP pool, ensuring even distribution across all chromosomes for Sanger sequencing validation. Genomic DNA from two Yangtze finless porpoise individuals was used as the template. Primer pairs flanking each candidate SNP (150 bp upstream and downstream) were designed using Primer Premier 5.0 (Supplementary Table S1). Polymerase chain reaction (PCR) was performed in a 20 μL reaction mixture containing 1 μL of DNA template, 0.5 μL of each forward and reverse primer (10 μM), 10 μL of 2× Taq PCR Master Mix (Vazyme Biotech Co., Ltd., Nanjing, China), and 7 μL of ddH_2_O. The thermal cycling protocol was as follows: initial denaturation at 95 °C for 5 min; 30 cycles of denaturation at 95 °C for 30 s, annealing at 58 °C for 30 s, and extension at 72 °C for 1 min; followed by a final extension at 72 °C for 10 min. Amplification success was confirmed by visualizing clear and specific bands on agarose gel electrophoresis. Subsequently, PCR products yielding high-quality sequencing chromatograms with single, unambiguous peaks at the target SNP position were considered successfully validated and retained for downstream KASP genotyping.

### 2.5. KASP Genotyping

Based on the candidate SNP loci identified, flanking sequences (150 bp upstream and downstream) were extracted for KASP assay design. Primer sets were designed using Primer Premier 5.0 (Supplementary Table S2). Each KASP primer set consisted of three primers: two Kompetitive allele-specific forward primers (F1 and F2) and one common reverse primer (R). The allele-specific forward primers were synthesized with the standard KASP fluorescent tails: the F1 primer carried the 5′ FAM tail (5′-GAAGGTGACCAAGTTCATGCT-3′), while the F2 primer carried the 5′ VIC tail (5′-GAAGGTCGGAGTCAACGGATT-3′). All primers were synthesized by Nanjing Genepioneer Biotechnology Co., Ltd. (Nanjing, China).

Genomic DNA was extracted from 28 Yangtze finless porpoise tissue samples using the QIAGEN DNeasy Blood & Tissue Kit (QIAGEN GmbH, Hilden, Germany) and served as the template for downstream analyses. All KASP primers were resuspended and adjusted to a working stock concentration of 10 μM in TE buffer (pH 8.0). The upstream primers F1 and F2 were mixed with the downstream universal primer R at a volume ratio of 1:1:3 to prepare a KASP primer premix, which was subsequently diluted to a working concentration of 0.1 μM. The PCR was performed in a total volume of 5 μL, consisting of 1.25 μL DNA template, 2.5 μL of 2× KASP Master Mix (LGC Genomics Ltd., Hoddesdon, UK), and 1.25 μL of the diluted primer pre-mix. Following sample loading, the PCR plate was sealed with an adhesive film, vortexed briefly, and centrifuged to ensure complete mixing of the reaction mixture prior to PCR amplification. The touchdown PCR protocol, featuring a stepwise reduction in annealing temperature from 61 °C to 55 °C, was implemented to enable specific amplification of SNP loci and accurate dual-allele genotyping. The detailed amplification conditions for the KASP–PCR reaction are provided in Supplementary Table S3. Following completion of the amplification process, fluorescence signals were acquired using the Bio-Rad CFX Connect real-time PCR system (Bio-Rad, Hercules, CA, USA), and genotyping results for each sample were subsequently determined.

### 2.6. Validation of SNP Effectiveness

To validate the effectiveness of the developed polymorphic SNP markers, 30 individuals from the Poyang Lake natural population were genotyped using the KASP assay. The resulting genotype data for all samples were analyzed with GenAlEx 6.5 [36] to calculate key genetic diversity parameters, including number of alleles (Na), effective number of alleles (Ne), Shannon–Wiener Index (I), observed heterozygosity (Ho), expected heterozygosity (He), inbreeding coefficient (F_IS_), major allele frequency (MAF), and polymorphism information content (PIC). The PIC for each locus was calculated according to the formula established by Botstein et al. [37]:

$$\mathrm{PIC}\;=\;1\;-\;\sum_{i=1}^{n}P_{i}^{2}\;-{\;\sum }_{i=1}^{n-1}{\;\sum }_{j=i+1}^{n}\;2P_{i}^{2}P_{j}^{2}$$

In the formula, n denotes the total number of alleles, while P_i_ and P_j_ represent the frequencies of the i-th and j-th alleles, respectively.

## 3. Results

### 3.1. Polymorphic SNP Marker Screening

The genome-wide variant discovery and filtering pipeline yielded an initial set of 29,177 candidate SNP loci, which showed relatively even chromosomal distribution (Figure 2). To ensure broad genomic coverage for downstream validation, a subset of 1070 SNPs evenly distributed across all chromosomes was randomly selected (Supplementary Figure S2). From this subset, 50 SNPs located on autosomes and the X chromosome were further chosen, guided by the principle of even chromosomal representation, for experimental validation via Sanger sequencing (Supplementary Table S1). The validation results confirmed the reliability of our in silico screening approach. Of the 50 candidate SNPs subjected to Sanger sequencing, 35 (70%) were successfully validated, exhibiting clear single-peak chromatograms at the target position, and were thus retained as true polymorphic sites. Representative agarose gel electrophoresis images and sequencing chromatograms for these validated SNPs are provided in Supplementary Figures S3 and S4, respectively. The 15 excluded loci failed primarily due to ambiguous sequencing peaks or unsuccessful PCR amplification, likely attributable to localized complex genomic regions. These 35 validated SNPs served as the final candidate panel for high-throughput genotyping using the KASP assay (Supplementary Figure S1).

### 3.2. KASP Genotyping Validation

**Figure 3:**
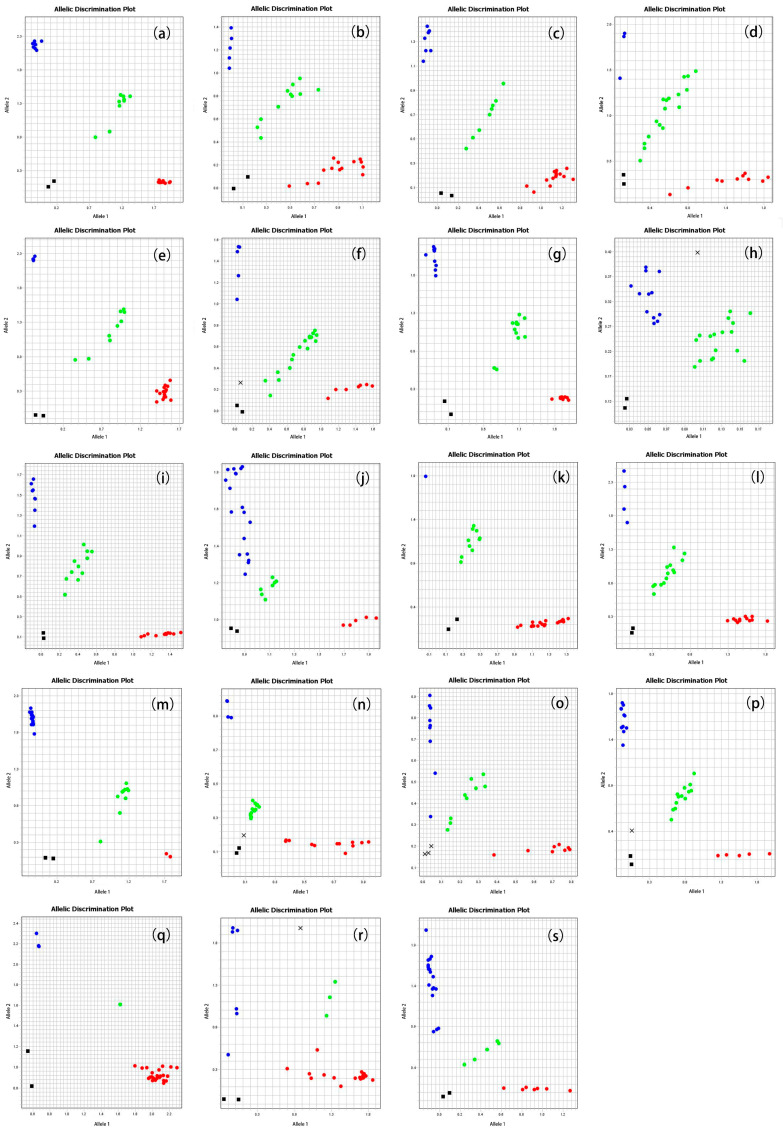
KASP genotyping results for the 19 validated polymorphic SNP loci. Each scatter plot (**a**–**s**) represents one SNP locus, showing the genotype clusters for 28 samples. Homozygous genotypes for the FAM and VIC alleles are plotted near the X-axis (red) and Y-axis (blue), respectively. Heterozygous genotypes (green) are distributed along the diagonal. The no-template control (NTC) is indicated by a black square near the origin.

The panel of 35 Sanger-validated SNPs was subjected to high-throughput genotyping using the KASP assay across 28 Yangtze finless porpoise individuals. KASP primers were successfully designed for all 35 loci (Supplementary Table S2), demonstrating the feasibility of transitioning these markers to a high-throughput platform. Among the 35 SNPs, 32 (91.43%) were successfully genotyped across all 28 samples, exhibiting clear cluster separation in the allele discrimination plot. The three failed loci (Snp01, Snp38, Snp43) showed ambiguous or nonspecific amplification, likely due to suboptimal primer specificity or the presence of nearby genomic variations interfering with assay performance. Of the 32 successfully genotyped SNPs, 19 (59.38% of the genotyped set, or 54.29% of the initial 35) were confirmed to be polymorphic (Figure 3 and Table 1).

### 3.3. Validation of Polymorphic SNP Loci

Genetic diversity was assessed across the 19 polymorphic SNP loci using 30 individuals from the Poyang Lake population of the Yangtze finless porpoise. The KASP genotyping results for all individuals are presented in Supplementary Figure S5 and Table S4, demonstrating clear allele clustering and high assay reliability. All loci were biallelic, yielding a total of 38 alleles. As summarized in Table 2, the effective number of alleles (Ne) ranged from 1.342 to 1.998 (mean = 1.791), while the Shannon–Wiener index (I) varied between 0.423 and 0.693 (mean = 0.625). The observed heterozygosity (Ho) spanned from 0.033 to 0.586 (mean = 0.350), and the expected heterozygosity (He) ranged from 0.255 to 0.497 (mean = 0.435). The inbreeding coefficient (F_IS_) showed considerable variation across loci, ranging from −0.415 to 0.869 (mean = 0.209). Major allele frequency (MAF) values ranged from 0.517 to 0.850 (mean = 0.653), and polymorphism information content (PIC) varied from 0.222 to 0.375 (mean = 0.338). Notably, the distribution of PIC values revealed that 47.37% of the loci (9/19) exhibited moderate polymorphism (0.25 ≤ PIC < 0.35), while the remaining 52.63% (10/19) showed high polymorphism (0.35 ≤ PIC ≤ 0.5). Additionally, except for three loci (Snp46, Snp47, and Snp48, *p*< 0.05), the remaining 16 loci conformed to Hardy–Weinberg equilibrium (*p* > 0.05), indicating good genetic stability within the tested population. These findings collectively demonstrate that the developed SNP markers possess moderate-to-high levels of genetic polymorphism and overall genetic equilibrium, confirming their utility for subsequent population genetic studies.

## 4. Discussion

This study successfully developed an efficient SNP marker system for the Yangtze finless porpoise by integrating a chromosome-level genome assembly, whole-genome resequencing, and Kompetitive Allele-Specific PCR (KASP) genotyping technology. The integrated approach addresses the critical need for high-throughput and reliable molecular markers in the conservation genetics of this endangered species. By leveraging genomic data for precise locus identification and employing KASP technology for high-throughput experimental validation, the system significantly enhances the efficiency and reliability of SNP marker development.

Through this development system, the relatively abundant SNP density observed on the Y chromosome in our dataset indicates potential genomic or evolutionary characteristics specific to the Yangtze finless porpoise. Y-chromosome markers serve as valuable tools in population genetics, particularly for tracing male-mediated gene flow and conducting sex-biased dispersal analyses [38,39]. The development of Y chromosome-specific SNP or STR markers in Yangtze finless porpoise will facilitate future applications, such as geographic origin tracing of deceased individuals and the assessment of threat-related factors in wild populations.

A total of 35 authentic polymorphic SNP loci were successfully validated and retained from an initial set of 50 candidates, achieving a validation success rate of 70%. This rate is significantly higher than the 60% retention rate reported by Li et al. [23] using a whole-genome shotgun sequencing strategy. The improved screening efficiency can be attributed to three key methodological advancements. First, the integration of a chromosome-level, high-quality reference genome with whole-genome resequencing data from multiple individuals not only provided a continuous and accurate genomic framework for sequence alignment but also enabled the systematic identification of genuine polymorphic loci across the entire genome [40,41]. This approach substantially enhanced the population representativeness and polymorphism potential of candidate SNPs [42,43]. Second, stringent bioinformatics filtering criteria were applied during candidate selection, including the retention of SNP loci with quality scores greater than 1000 and polymorphism information content (PIC) values ranging from 0.2 to 0.5, thereby effectively eliminating low-quality and weakly polymorphic variants. Third, experimental validation of candidate loci was performed via Sanger sequencing, which further confirmed their authenticity and polymorphic status. Collectively, these improvements facilitated the efficient identification of high-quality, highly polymorphic SNP markers, providing robust molecular tools for population genetic studies of the Yangtze finless porpoise.

In the subsequent validation phase, KASP technology was employed to validate the specificity of the screened SNP loci, resulting in the successful development of 19 polymorphic SNP markers, representing 54.29% of the initial candidate set. This outcome highlights the dual advantages of integrating genomic screening with KASP-based specificity validation. Previous efforts to develop SNPs in *Neophocaena*, such as the study by Li et al. [24] involved multiple labor-intensive steps: shotgun library construction, cross-species comparative anchor-tagged sequencing (CATS) primer screening, and genotyping via fragment length differential allele-specific PCR (FLDAS-PCR). While that approach yielded 140 candidate SNPs, the workflow was complex and resource-demanding. In contrast, the strategy presented here integrates chromosome-level genomes directly with the KASP genotyping platform. This integrated approach significantly improves efficiency, enhances reproducibility, and reduces costs, making it better suited for large-scale population genetic monitoring and analysis. The efficiency of KASP validation stems from its allele-specific primer design, in which precise complementarity at the 3′-end base enables targeted verification of candidate loci. This selective amplification mechanism, combined with fluorescence resonance energy transfer (FRET) detection, effectively discriminates against nonspecific amplification arising from primer mismatches, thereby providing experimental confirmation of both the authenticity and polymorphism of the candidate SNPs [44,45]. In comparison to previous studies that utilized high-throughput methods such as RAD-seq to screen larger numbers of candidate loci—such as 105 SNPs in the Chinese softshell turtle [46] and 24 SNPs in the yellow catfish [47]—the strategy implemented in this study yielded a smaller number of final markers but ensured that each retained locus exhibits clear allele-specific genotyping capability and consistent amplification efficiency through rigorous KASP validation. This specificity-driven screening approach effectively eliminates false-positive loci originating from sequencing errors, paralogous sequence interference, or repetitive genomic regions, thereby substantially improving the validity and practical utility of the developed markers [48]. The methodology aligns with that used in giant panda research, where 19 SNP markers were successfully established by integrating genomic data with KASP technology, underscoring the pivotal role of KASP in transforming high-throughput genomic candidates into stable, reliable, and scalable molecular tools.

Polymorphism Information Content (PIC) is a commonly used indicator to measure the polymorphism of molecular markers [49]. The higher the PIC value of genetic molecular markers, the richer the genetic information they contain. According to the standard proposed by Botstein et al. [37], when the PIC value is between 0.25 and 0.5, the genetic polymorphism of genetic molecular markers is classified as moderate level [50]. Nineteen polymorphic SNP markers developed in this study exhibited high levels of genetic diversity within the Poyang Lake population of Yangtze finless porpoises. Analyses revealed that both observed heterozygosity (Ho = 0.350) and expected heterozygosity (He = 0.435) were significantly higher than those reported by Li et al. [23] for SNP markers screened in the porpoise genus using comparative anchor tagged sequencing (CATS) and fragment length differential allele-specific PCR (FLDAS-PCR) (Ho = 0.187, He = 0.248). These values also exceeded those reported by Li et al. [24] for Yangtze finless porpoise SNP markers developed via whole-genome shotgun sequencing combined with CATS (Ho = 0.292, He = 0.340). These results indicate that the SNP loci selected in this study possess superior polymorphism and are more effective at capturing population genetic variation. This advantage is primarily attributable to the integration of genomic data-based screening with KASP validation, which ensures that the final markers retain high polymorphic potential while demonstrating robust experimental stability and practical utility [50]. As a result, these markers provide more reliable molecular tools for population genetic studies of the Yangtze finless porpoise.

## 5. Conclusions

The polymorphic SNP marker system developed in this study demonstrates considerable application potential. While preliminary results from the pilot sample set are promising, comprehensive validation across broader geographical and demographic samples is required to fully assess its efficiency. To this end, we have expanded sample collection to include multiple geographic populations across the middle and lower reaches of the Yangtze River and its associated lakes. Once a sufficient sample size is obtained, we will apply standard analytical approaches—such as population structure analysis and genetic differentiation assessment—to verify the marker panel’s capacity for detecting spatial heterogeneity and quantifying genetic divergence. The resulting validation data will establish a scientific foundation for linking these genetic insights to targeted, evidence-based conservation measures and regional management strategies. Upon thorough validation, this marker set is expected to provide reliable technical support for genetic diversity assessment and kinship analysis in Yangtze finless porpoise conservation programs. Furthermore, the methodological framework developed in this study may serve as a reference for conservation genetics research in other endangered species.

## Figures and Tables

**Table 1:** Genomic characteristics of the 19 validated polymorphic SNP loci.

N	Locus ID	Genomic Position	Variant Type	N	Locus ID	Genomic Position	Variant Type
1	Snp03	Chr02_3578746	C/T	11	Snp30	Chr14_13332465	A/G
2	Snp04	Chr02_90116171	A/G	12	Snp31	Chr15_1462494	T/G
3	Snp08	Chr04_6297226	A/G	13	Snp32	Chr16_55868804	G/A
4	Snp09	Chr04_71791661	T/C	14	Snp34	Chr17_23473466	A/C
5	Snp12	Chr04_84395785	A/G	15	Snp35	Chr17_66317367	T/G
6	Snp14	Chr04_106682173	G/A	16	Snp36	Chr18_33806104	G/A
7	Snp19	Chr07_50780006	G/C	17	Snp46	ChrX_31038355	T/C
8	Snp20	Chr07_85509514	C/A	18	Snp47	ChrX_33057094	C/T
9	Snp25	Chr11_27764344	G/A	19	Snp48	ChrX_37770280	G/T
10	Snp29	Chr13_83891862	T/C				

**Table 2:** Population genetic diversity indices for Yangtze finless porpoises in Poyang Lake based on 19 SNP markers.

Locus ID	Na	Ne	I	Ho	He	F_IS_	MAF	PIC	HWE
Snp03	2	1.946	0.679	0.300	0.486	0.383	0.583	0.368	NS
Snp04	2	1.835	0.647	0.367	0.455	0.194	0.650	0.351	NS
Snp08	2	1.923	0.673	0.267	0.480	0.444	0.600	0.365	NS
Snp09	2	1.897	0.666	0.567	0.473	−0.199	0.617	0.361	NS
Snp12	2	1.684	0.596	0.300	0.406	0.261	0.717	0.324	NS
Snp14	2	1.991	0.691	0.586	0.498	−0.178	0.534	0.374	NS
Snp19	2	1.998	0.693	0.433	0.499	0.132	0.517	0.375	NS
Snp20	2	1.708	0.605	0.586	0.414	−0.415	0.707	0.329	NS
Snp25	2	1.980	0.688	0.367	0.495	0.259	0.550	0.372	NS
Snp29	2	1.684	0.596	0.233	0.406	0.425	0.717	0.324	NS
Snp30	2	1.471	0.500	0.333	0.320	−0.042	0.800	0.269	NS
Snp31	2	1.867	0.657	0.467	0.464	−0.005	0.633	0.357	NS
Snp32	2	1.557	0.543	0.333	0.358	0.068	0.767	0.294	NS
Snp34	2	1.859	0.655	0.448	0.462	0.030	0.638	0.355	NS
Snp35	2	1.989	0.690	0.333	0.497	0.330	0.537	0.374	NS
Snp36	2	1.918	0.672	0.448	0.479	0.063	0.603	0.364	NS
Snp46	2	1.342	0.423	0.033	0.255	0.869	0.850	0.222	*
Snp47	2	1.622	0.572	0.103	0.383	0.730	0.741	0.310	*
Snp48	2	1.763	0.624	0.167	0.433	0.615	0.683	0.339	*
Mean	2	1.791	0.625	0.350	0.435	0.209	0.653	0.338	

Note: Na, number of alleles; Ne, effective number of alleles; I, Shannon–Wiener index; Ho, observed heterozygosity; He, expected heterozygosity; F_IS_, inbreeding coefficient; MAF, major allele frequency; PIC, polymorphism information content; HWE, Hardy–Weinberg Equilibrium. NS indicates no significant difference (*p* > 0.05), * indicates significant difference (*p* < 0.05).

## Data Availability

The original contributions presented in this study are included in the article/Supplementary Material. Further inquiries can be directed to the corresponding authors.

## Supplementary Materials

The following supporting information can be downloaded at: https://www.mdpi.com/article/10.3390/ani16030475/s1, Figure S1: Technical roadmap for the development of polymorphic SNP markers of the Yangtze finless porpoise; Figure S2: Distribution of the 1070 candidate SNPs selected for downstream analysis across all chromosomes; Figure S3: Representative agarose gel electrophoresis images for validated SNPs; Figure S4: Representative sequencing chromatograms for validated SNPs; Figure S5: KASP genotyping for Yangtze finless porpoises in Poyang Lake based on 19 SNP markers; Table S1: Primer sequences used for Sanger sequencing validation of candidate SNP loci; Table S2: Primer sequences used for KASP assay design and validation of 35 SNP loci; Table S3: KASP Competitive Allele-Specific PCR Amplification Reaction Conditions; Table S4: KASP genotyping for Yangtze finless porpoises in Poyang Lake based on 19 SNP markers.
