# Supplementary material for: Genome-Wide Development and Validation of KASP-Based SNP Markers in Neophocaena asiaeorientalis asiaeorientalis
Source: Animals (Basel). 2026 Feb 3;16(3):475. doi: 10.3390/ani16030475 (PMC12896827; doi:10.3390/ani16030475)
Supplement: Supplementary file 1 [file animals-16-00475-s001.zip › animals-4070898-supplementary.pdf]

Supplementary Materials

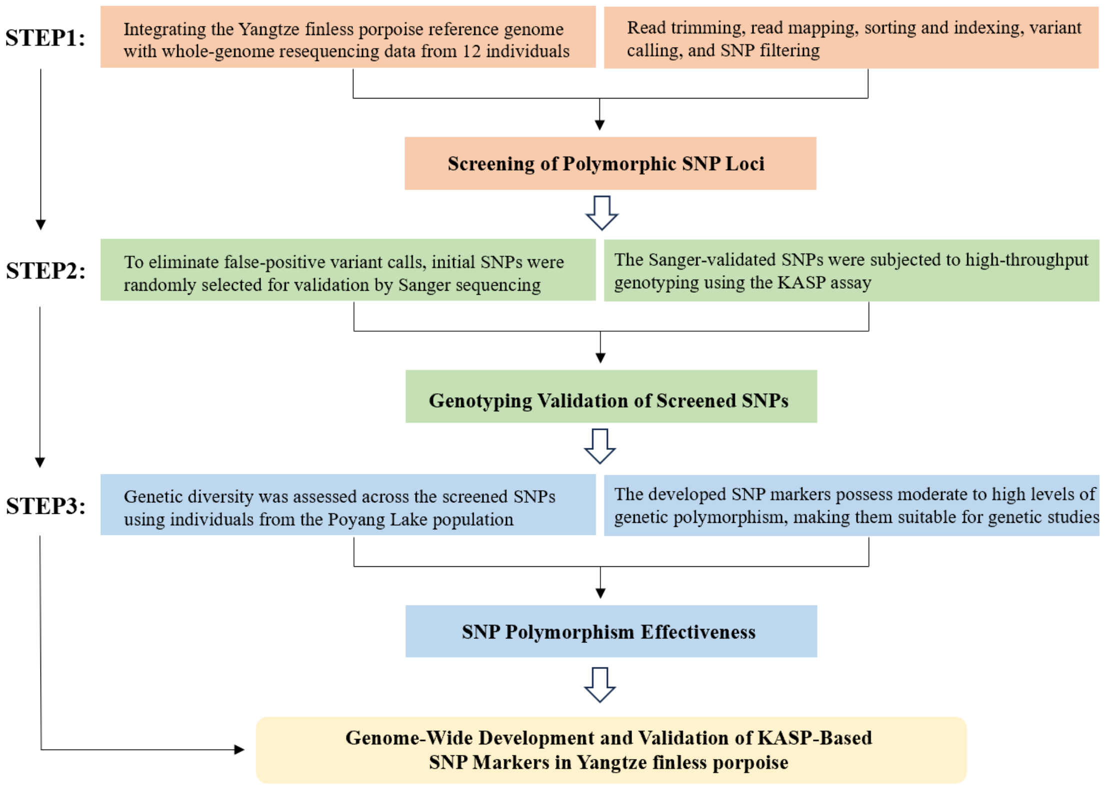

Supplementary Figure S1. Technical roadmap for the development of polymorphic SNP markers of the Yangtze finless porpoise.

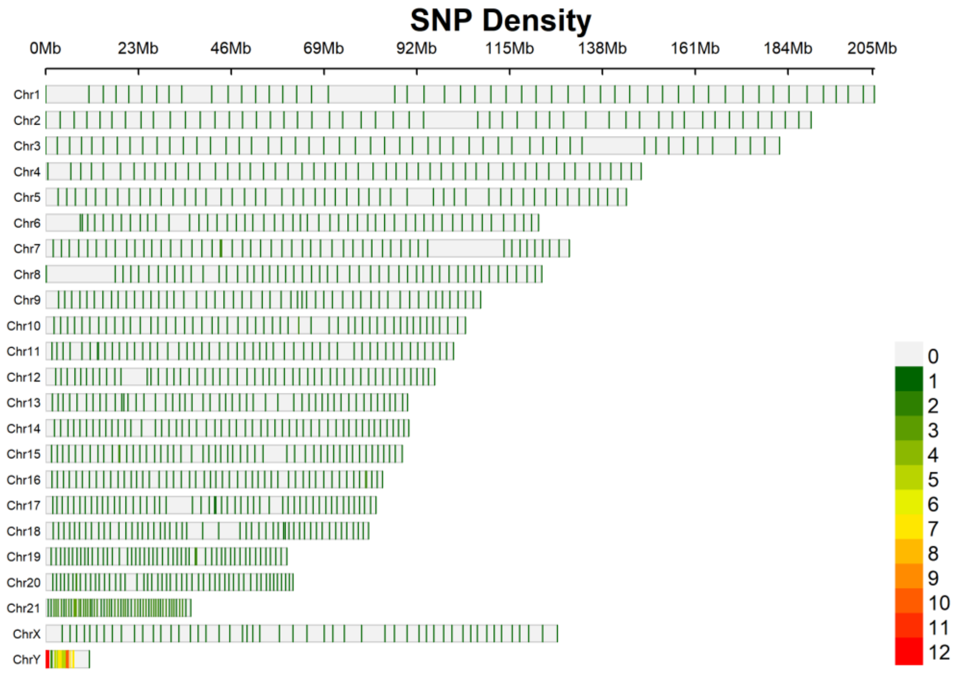

Supplementary Figure S2. Distribution of the 1,070 candidate SNPs selected for downstream analysis across all chromosomes.

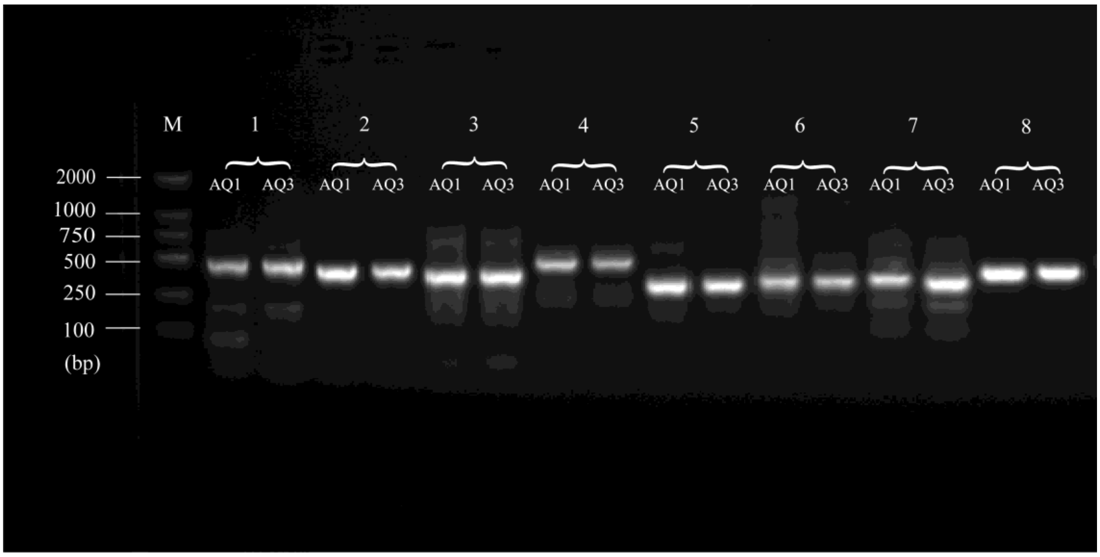

Supplementary Figure S3. Representative agarose gel electrophoresis images for validated SNPs. 1. Locus Snp25 (Chr11_27764344); 2. Locus Snp24 (Chr10_84082545); 3. Locus Snp26 (Chr11_7646940); 4. Locus Snp28 (Chr13_57538364); 5. Locus Snp29 (Chr13_83891862); 6. Locus Snp32 (Chr16_55868804); 7. Locus Snp36 (Chr18_33806104); 8. Locus Snp38 (Chr19_45792533).

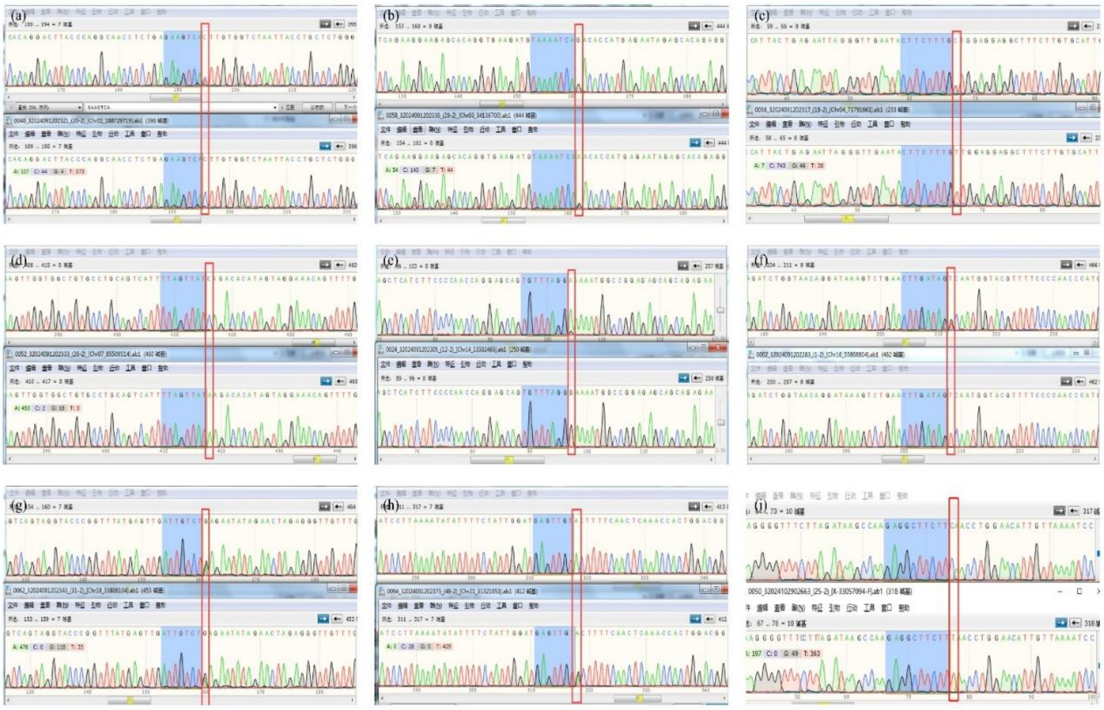

Supplementary Figure S4. Representative sequencing chromatograms for validated SNPs. (a). Locus Snp01 (Chr01_188729719); (b). Locus Snp06 (Chr03_34136700); (c). Locus Snp09 (Chr04_71791661); (d). Locus Snp20 (Chr07_85509514); (e). Locus Snp30 (Chr14_13332465); (f). Locus Snp32 (Chr16_55868804); (g). Locus Snp36 (Chr18_33806104); (h). Locus Snp43 (Chr21_31321051); (i). Locus Snp47 (Chr04_6297226).

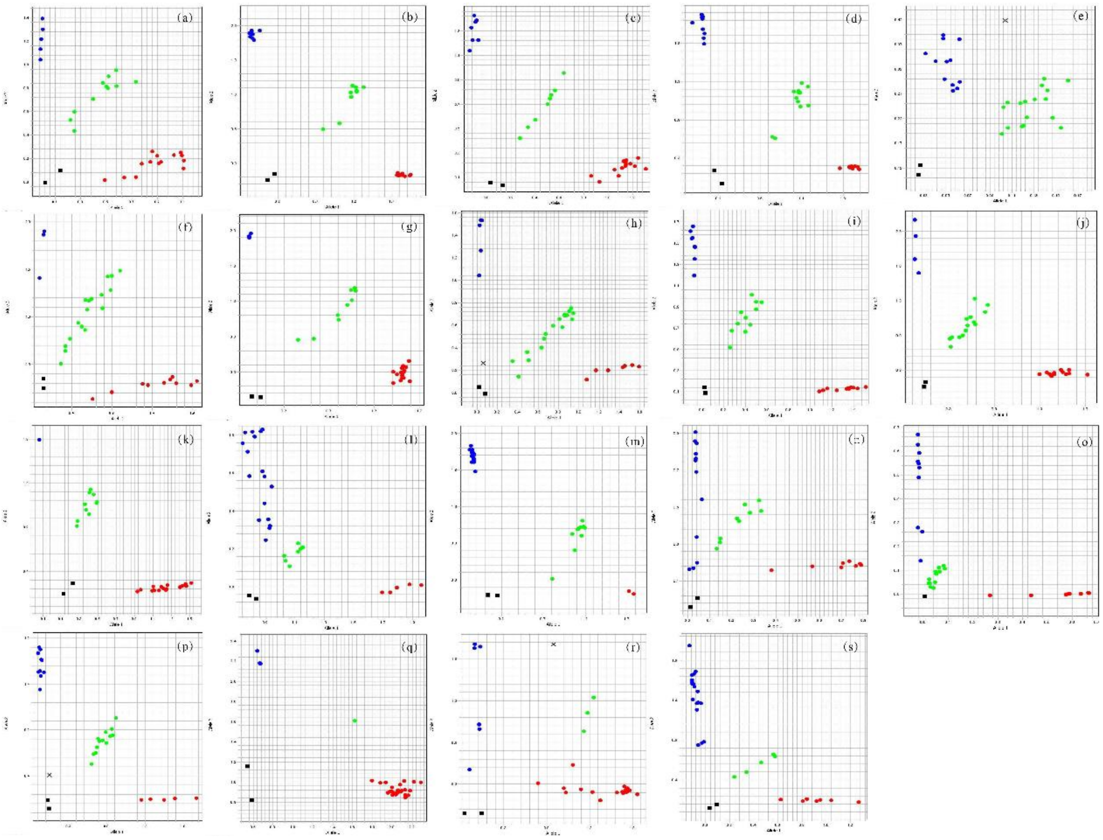

Supplementary Figure S5. KASP genotyping for Yangtze finless porpoises in Poyang Lake based on 19 SNP markers. Each scatter plot (a to s) represents one SNP locus, showing the genotype clusters for 30 samples. Homozygous genotypes for the FAM and VIC alleles are plotted near the X-axis (red) and Y-axis (blue), respectively. Heterozygous genotypes (green) are distributed along the diagonal. The no-template control (NTC) is indicated by a black square near the origin.

Supplementary Table S1. Primer sequences used for Sanger sequencing validation of candidate SNP loci.

Locus ID	Genomic position	Forward primer F (5'-3')	Reverse primer R (5'-3')
Snp01	Chr01_188729719	CTGTCTTCCTCTCTCCGTGG	GTGCAGACACACACAAACCC
Snp02	Chr01_202624202	GTGGGCAACCTTGTCTTGTT	GCAAACAGAATCCAACAGCA
Snp03	Chr02_3578746	GAGTGGGTGGGGTCCTTATT	ACCCAGGACTGACTGTCACC
Snp04	Chr02_90116171	CTGGGTTGTGTTCATGGTTG	TTTGAGATTGTGGAGAGGGG
Snp05	Chr02_179985313	CCACTTGGACTCCAGGCTTA	CTCCCCACAGATCAGCAAAT
Snp06	Chr03_34136700	GGCTCTGTATGGCAAATGGT	CACAGCAAAATGGAGCTGAA
Snp07	Chr03_40839176	AGAGTTGGTGCAGACAGCCT	TAATTCCCCAAGACACCCAA
Snp08	Chr04_6297226	CATGCGCGAACTCTCAATTA	CTGGATGGCTGATGATGTTG
Snp09	Chr04_71791661	AGGAGTTGCAAGAGGCAAAA	TCGAGAGACATTTGGCAGTG
Snp10	Chr04_6297226	CATGCGCGAACTCTCAATTA	CTGGATGGCTGATGATGTTG
Snp11	Chr04_71791661	AGGAGTTGCAAGAGGCAAAA	TCGAGAGACATTTGGCAGTG
Snp12	Chr04_84395785	AGGCAGGTGTTTTTGGTTTG	ACCTTTAGGACCTGGGCAGT
Snp13	Chr04_89535712	GGTGTCTCTGATGCATTCCA	GGTGTCTCTGATGCATTCCA
Snp14	Chr04_106682173	TATGGAAGGCCCTGTCATTG	TTTCCTTCTGGGAAGAGGCT
Snp15	Chr05_52030847	ACAAAGCACCGAGCTGATCT	AGCCTGGAGATGGAAACAAC
Snp16	Chr05_69137087	TCATTCCTTTGCAGGTGGAT	TGTCAGTACTGCCCAAAGCA
Snp17	Chr06_67713017	GCCACTCTCTCACTTCGTCC	CATACGACTCAGCAATCCCA
Snp18	Chr07_36322501	GGGCAGAACCATAGGACAAA	TGCATCTGTACCCCTCCTTC
Snp19	Chr07_50780006	ATGCAGGTTCCAACCTATGC	CCATGACCAATTCATCACCA
Snp20	Chr07_85509514	TGCAACGCATTACCTAGCTG	AATGGCTGAAAATCCCAAAA
Snp21	Chr08_34077431	TACCACAACTTCCTGGAGCC	TTACTCAGCCGTAAAACGGG
Snp22	Chr09_55597512	GGTTTTATTTGGGAGAGGGG	CCAGCCACAGTCTTACCCAG
Snp23	Chr10_72524451	CTTCGCCCTTCATGTCAGTT	GCAGAAAGGCTCAGGTATGG
Snp24	Chr10_84082545	TGGAGGGAGAGTGATCAACA	CATTGGAAGGGCTGTGTCTT
Snp25	Chr11_27764344	TCCCGTAGACAGTAGGTGGC	AAATGTCTGATGTTCCCCCA
Snp26	Chr11_7646940	GAACCTGTGTCCCCTGTGTT	CAAGCCAGAAGAGAGTGGCT
Snp27	Chr12_90501540	GAATTCAGCACCACCAAACC	AGGCTTGATTTGTGACCCAG
Snp28	Chr13_57538364	TTCAGTCAACCAGTTGTGCC	TCCAATTTCCCCATTTCTCA
Snp29	Chr13_83891862	CGACCGACTCAGAAGTGACA	ATTTGATCATCACCCCCAAA
Snp30	Chr14_13332465	AGAGCCTCACACCCTTCTCA	GTCAGCCGGACATGAAAGAT
Snp31	Chr15_1462494	TCCAATACCTGTGTGCGTGT	AGGGTCTGGTTTCCAGAGGT
Snp32	Chr16_55868804	AAAGCCCTAGTTCCGTCCAT	CCGGCAGAGTTAGAGACCAG
Snp33	Chr16_60186222	GAGGGAAGTCCAAAAGGGAG	TAGGAGGAGGGAGAAGGGAA
Snp34	Chr17_23473466	AGGTCCAGACAACTTCACCAG	CAACCTGGCATTGCTAAAAA
Snp35	Chr17_66317367	TCCCACAGGTTAGGCAAATC	TATTCCAGGCTTTGCCAGTT
Snp36	Chr18_33806104	ACTCTGTCTGGGAATGGGTG	CCACATGCCACAATATGAGC
Snp37	Chr19_7774549	GCTAGAGGGCATAGTGCAGG	GTGGTTTGGACTCCATGCTT
Snp38	Chr19_45792533	GCTTGTAGACGCATTGTCCA	CAGAAAAGACGGCCCTAAAA

Snp39	Chr19_49565046	TTTCATTCCCCTCCAAACTG	CCATGCTCCTCAGCAGTACA
Snp40	Chr20_5657632	AAAAATCAACCCTCTGGGCT	ATGGACACTTGGGTTGCTTC
Snp41	Chr20_36256950	CGTATCATGTGCCACACTCC	TATGGCGTTCTGCTTTGTTG
Snp42	Chr21_13798255	GTGTTTTGTGGTCCTTGCCT	GGGAGGGAGGTTATCTGCTC
Snp43	Chr21_31321051	TAACTGCTTCCGGTTCTTGG	TCCTTTACTGGTCACCCCAG
Snp44	ChrX_14498266	CGTGAGATGGCACAGTGAGT	CCATGAAACCATCACCACAA
Snp45	ChrX_26758248	GTGGGAAATGCACTCCAAGT	TAAAATGGGTCTTTGCTGGC
Snp46	ChrX_31038355	GCACAAATTTTGGTTGCCTT	AATTACACCTCCCTGGACCC
Snp47	ChrX_33057094	CCAATTTTCCTTTGAGGGTG	GTGCTCAATCAGTGTTCCCA
Snp48	ChrX_37770280	TGTACATTCCATGAGGGCAA	AGGGTGCTAGGTGCTGAGAA
Snp49	ChrX _42954445	GGGTTGTCTTCAACCTGCAT	TGCAGGAAGAGACCCTCACT
Snp50	ChrX_84088207	TTTCAGCACTGCCTTTGTTG	TCACTTTGCTGTACGCCTGAA

Supplementary Table S2. Primer sequences used for KASP assay design and validation of 35 SNP loci.

Locus ID	Forward primer F1 (5'-3')	Forward primer F2 (5'-3')	Reverse primer R (5'-3')
Snp01	GAAGGTGACCAAGTTCATGCTCAGGCAACCTCTGAGAAGTCAT	GAAGGTCGGAGTCAACGGATTCAGGCAACCTCTGAGAAGTCAC	GCCCCAGAGCAGGTAATTAGAC
Snp03	GAAGGTGACCAAGTTCATGCTGCTGCAGCCACATTTCCAC	GAAGGTCGGAGTCAACGGATTGCTGCAGCCACATTTCCAT	CGTAGGACCAGAGGGACCCT
Snp04	GAAGGTGACCAAGTTCATGCTTGGAACTCATAGCAGCAGGTAA	GAAGGTCGGAGTCAACGGATTTGGAACTCATAGCAGCAGGTAG	GTAAAGAATAAATGTAGGACAGAAACAG
Snp05	GAAGGTGACCAAGTTCATGCTGTGGAGGGCTTAATCTTGTCTTCAA	GAAGGTCGGAGTCAACGGATTGTGGAGGGCTTAATCTTGTCTTCAG	GGTTTACTGATGCTTCAATGCCTT
Snp06	GAAGGTGACCAAGTTCATGCTGAAGAGCACAGGTGAAGATGTAAAATCAG	GAAGGTCGGAGTCAACGGATTGAAGAGCACAGGTGAAGATGTAAAATCAA	CAGATTAGTTTCACTGCTCTCTAAACC
Snp08	GAAGGTGACCAAGTTCATGCTTCCACTTAAAGCAAACCTTACACA	GAAGGTCGGAGTCAACGGATTCCACTTAAAGCAAACCTTACACG	TCAATTTATTCTGTAAATACAAACGCT
Snp09	GAAGGTGACCAAGTTCATGCTGAGAATTAGGGTTGAATACTTCTTTGT	GAAGGTCGGAGTCAACGGATTGAATTAGGGTTGAATACTTCTTTGC	ACATGCTGCAATGCACAAGA
Snp12	GAAGGTGACCAAGTTCATGCTGATGCTGGGGAGTGGAAGA	GAAGGTCGGAGTCAACGGATTATGCTGGGGAGTGGAAGG	TTTTTCCACCACCTCTATGCTT
Snp13	GAAGGTGACCAAGTTCATGCTCAAGAATTAGACAATGGTGGAAGTCATGG	GAAGGTCGGAGTCAACGGATTCAAGAATTAGACAATGGTGGAAGTCATGA	CTATGAGTTCCATCACTGACTCTCTTTTC
Snp14	GAAGGTGACCAAGTTCATGCTTTCTGTTTTAGACAACATACTAGTCAATG	GAAGGTCGGAGTCAACGGATTATTCTGTTTTAGACAACATACTAGTCAATA	GGTTCTGGAACTGTCTTATACATCC
Snp16	GAAGGTGACCAAGTTCATGCTCATATATGCAAAGGTTTATTTCTGGGT	GAAGGTCGGAGTCAACGGATTCATATATGCAAAGGTTTATTTCTGGGC	ACAATAATTAAAACAGTGTAATATGGCAT
Snp19	GAAGGTGACCAAGTTCATGCTTATGCACTCACTTGCTCTCTCATG	GAAGGTCGGAGTCAACGGATTATATGCACTCACTTGCTCTCTCATC	GGAGAGTGTCCATACTGGGTGAA
Snp20	GAAGGTGACCAAGTTCATGCTTGCCTGCAGTCATTTTAGTTATC	GAAGGTCGGAGTCAACGGATTGTGCCTGCAGTCATTTTAGTTATA	CCAAAACTGTTTCCTACTATGTGTC

Snp23	GAAGGTGACCAAGTTCATGCTGAAGGAGAAAATGAGGCTCACAC	GAAGGTCGGAGTCAACGGATTGAAGGAGAAAATGAGGCTCACAT	TTCATTTCTGGTTCTCAATATCTGC
Snp24	GAAGGTGACCAAGTTCATGCTAAAATTTTAGAAAAGCATTACTTAGAAAGCA	GAAGGTCGGAGTCAACGGATTAAAATTTTAGAAAAGCATTACTTAGAAAGCG	TATCTCTGAGAGTGAGATTATGGAAAGAC
Snp25	GAAGGTGACCAAGTTCATGCTCAGATCCACTGCTTTCCTGG	GAAGGTCGGAGTCAACGGATTGCAGATCCACTGCTTTCCTGA	GGCAGCAATGAGAGGCAA
Snp26	GAAGGTGACCAAGTTCATGCTTTTGGTGGTGCTAAATTCTCTTAAT	GAAGGTCGGAGTCAACGGATTTTTGGTGGTGCTAAATTCTCTTAAC	CCACAGAGAAATTAAAACCTTTACAG
Snp28	GAAGGTGACCAAGTTCATGCTAACAGTTGGAAACACTGGATTAAAC	GAAGGTCGGAGTCAACGGATTAACAGTTGGAAACACTGGATTAAAA	ATGACCTGGATTAGATTTTAGAGGTT
Snp29	GAAGGTGACCAAGTTCATGCTTTCAGCAAAAAGTGAAACTAGACATT	GAAGGTCGGAGTCAACGGATTTTCAGCAAAAAGTGAAACTAGACATC	AGAGAGCCAATATCTAGAAGTTCGG
Snp30	GAAGGTGACCAAGTTCATGCTCAACCAGGAGCAGTGTTTAGGA	GAAGGTCGGAGTCAACGGATTAACCAGGAGCAGTGTTTAGGG	CCTTCTCTGCTGCTCTCCG
Snp31	GAAGGTGACCAAGTTCATGCTTATTGACATTCGCTATCTCTACTGAT	GAAGGTCGGAGTCAACGGATTTATTGACATTCGCTATCTCTACTGAG	AGTAAAGCGCACATACAGGGA
Snp32	GAAGGTGACCAAGTTCATGCTGGGGAAAACGTACCATTGC	GAAGGTCGGAGTCAACGGATTTGGGGAAAACGTACCATTGA	CCTAGAGTATTACAGGGGTGAGATC
Snp34	GAAGGTGACCAAGTTCATGCTCAGTTGATCAGGAAGTATTATCCTTTT	GAAGGTCGGAGTCAACGGATTCAGTTGATCAGGAAGTATTATCCTTTG	AATATTAGCAAATTGAATCCAGCAA
Snp35	GAAGGTGACCAAGTTCATGCTGAAAATATTATACCTTTTTTATATCGCTT	GAAGGTCGGAGTCAACGGATTAAAATATTATACCTTTTTTATATCGCTG	TAAAGAAAATATTAGCAGATCGAGC
Snp36	GAAGGTGACCAAGTTCATGCTCCGGTTTATGAGTTGATTGTCTG	GAAGGTCGGAGTCAACGGATTCCCGGTTTATGAGTTGATTGTCTA	TGTGCAAACAACCCTCTAGTTCTAT
Snp38	GAAGGTGACCAAGTTCATGCTTTTATGTATAAAATTACAAATACTTTAGAAAAAAT	GAAGGTCGGAGTCAACGGATTTTATGTATAAAATTACAAATACTTTAGAAAAAAC	CATATTTCAACCCGTTACACTCTG
Snp42	GAAGGTGACCAAGTTCATGCTCGGAGTCTAAGTGAAGTTTTACACACCTA	GAAGGTCGGAGTCAACGGATTCGGAGTCTAAGTGAAGTTTTACACACCTG	CTATTGTAAGCATCTGCCGATTGACTC

Snp43	GAAGGTGACCAAGTTCATGCTAAAATATATTTTCTATTGGATGAGTTGTA	GAAGGTCGGAGTCAACGGATTAATATATTTTCTATTGGATGAGTTGTG	ACCGTCCAGTGGTTTGAGTT
Snp44	GAAGGTGACCAAGTTCATGCTGTATCCCCACTTCCACAAAGTT	GAAGGTCGGAGTCAACGGATTTATCCCCACTTCCACAAAGTG	CTGTACAGTTGATCTCTAGGACTTATTC
Snp45	GAAGGTGACCAAGTTCATGCTCAAAAACTGCCATTATCATGAAG	GAAGGTCGGAGTCAACGGATTCAAAAACTGCCATTATCATGAAA	AAGGTCAGACTTCTATCCCTAAATC
Snp46	GAAGGTGACCAAGTTCATGCTCTTTTCAAACCTCTGCTTGTGAT	GAAGGTCGGAGTCAACGGATTCTTTTCAAACCTCTGCTTGTGAC	TGACCAATGGGACATAAGCAAG
Snp47	GAAGGTGACCAAGTTCATGCTTTAGATAAGCCAAGAGGCTTCTTC	GAAGGTCGGAGTCAACGGATTTTAGATAAGCCAAGAGGCTTCTTT	TGGAGGATTTTAACAATGTTCCA
Snp48	GAAGGTGACCAAGTTCATGCTCTTCTGCTTGGAAGTGGCAG	GAAGGTCGGAGTCAACGGATTCTTCTGCTTGGAAGTGGCAT	GCTTTGGCCAATGAAATGTG
Snp49	GAAGGTGACCAAGTTCATGCTCAAAGTAGGTGATCTGCAGTTGGAT	GAAGGTCGGAGTCAACGGATTCAAAGTAGGTGATCTGCAGTTGGAC	CCTGTACACAATCTGTTTCCTTCC
Snp50	GAAGGTGACCAAGTTCATGCTGCTAATCCTATAGGCAAAATACCG	GAAGGTCGGAGTCAACGGATTTGCTAATCCTATAGGCAAAATACCA	TGATAAGAAAAATTAAAACAATACAGAAAC

Supplementary Table S3. KASP Competitive Allele-Specific PCR Amplification Reaction Conditions.

Step	Content	Temperature (°C)	Time (s)	Cycle Number
1	Initial Denaturation	95	600	1
2	Denaturation	95	20	10
	Annealing/Extension	61-55	60	
3	Denaturation	95	20	27
	Annealing/Extension	55	60	
4	Reading	25	30	1

Supplementary Table S4. KASP genotyping for Yangtze finless porpoises in Poyang Lake based on 19 SNP markers.

Sample	Locus ID																		
	Snp 03	Snp 04	Snp 08	Snp 09	Snp 12	Snp 14	Snp 19	Snp 20	Snp 25	Snp 29	Snp 30	Snp 31	Snp 32	Snp 34	Snp 35	Snp 36	Snp 46	Snp 47	Snp 48
YFP29	C:C	G:G	A:G	T:C	A:G	G:A	G:C	A:A	G:G	C:C	A:G	T:G	T:T	A:A	G:G	G:A	T:T	C:T	G:T
YFP30	C:C	A:G	A:G	T:C	A:A	G:A	G:G	A:A	G:A	C:C	A:G	T:G	G:T	A:C	T:G	A:A	T:T	C:C	G:G
YFP31	C:C	A:G	A:G	T:T	A:A	G:G	G:C	C:A	G:A	C:C	A:A	T:G	T:T	A:C	T:G	NA	T:T	C:C	G:T
YFP32	C:T	A:G	A:A	T:T	G:G	G:G	G:G	A:A	G:A	T:C	A:G	T:T	T:T	C:C	T:T	G:G	T:T	C:C	T:T
YFP33	C:C	A:A	A:A	T:T	A:A	G:G	C:C	C:A	A:A	C:C	A:A	T:T	T:T	A:A	T:T	A:A	T:T	T:T	G:G
YFP34	C:T	A:A	A:G	T:T	G:G	G:A	C:C	C:A	G:A	T:T	A:A	T:G	T:T	C:C	T:T	G:A	T:T	C:C	T:T
YFP35	C:T	A:G	G:G	T:C	A:G	G:A	C:C	C:A	G:G	T:C	A:A	T:G	T:T	A:C	NA	G:A	T:T	T:T	T:T
YFP36	C:C	A:G	G:G	C:C	A:A	G:G	G:C	C:A	G:G	C:C	A:G	T:G	G:T	A:C	NA	G:A	T:T	NA	T:T
YFP37	C:C	G:G	G:G	T:C	G:G	G:A	C:C	A:A	G:A	T:C	A:A	T:G	T:T	A:C	T:G	G:A	T:T	T:T	G:T
YFP38	T:T	A:A	G:G	T:T	A:A	G:A	C:C	C:A	G:A	T:C	A:A	T:T	T:T	A:A	T:G	A:A	C:C	C:C	T:T
YFP39	C:C	A:A	A:A	T:C	A:A	A:A	G:C	A:A	A:A	C:C	A:G	T:G	G:T	A:C	G:G	A:A	T:T	C:C	T:T
YFP40	C:C	A:A	A:A	T:C	A:G	A:A	G:C	A:A	G:G	C:C	A:A	T:T	T:T	A:A	T:T	G:A	T:T	C:C	T:T
YFP41	C:T	A:A	A:G	T:C	A:A	G:G	G:C	C:A	G:A	T:C	A:G	T:G	G:T	A:C	G:G	G:A	T:T	T:T	T:T
YFP42	T:T	A:A	A:G	T:C	G:G	G:A	C:C	C:A	G:A	C:C	A:A	T:T	T:T	A:C	T:G	A:A	T:T	C:C	T:T
YFP43	T:T	A:A	G:G	T:T	A:A	G:A	G:G	A:A	G:G	T:T	A:A	T:G	T:T	A:A	T:T	G:A	T:T	C:C	G:G
YFP44	C:T	A:A	A:A	T:T	A:A	G:A	C:C	A:A	G:A	C:C	A:G	T:G	G:T	C:C	NA	G:G	T:T	C:C	T:T
YFP45	C:C	A:A	G:G	C:C	A:G	G:G	G:C	C:A	A:A	T:C	A:G	G:G	T:T	A:A	T:G	G:A	T:T	C:C	G:G
YFP46	T:T	A:A	A:G	T:T	A:A	A:A	C:C	A:A	G:G	T:T	G:G	G:G	T:T	A:A	G:G	A:A	C:C	C:C	T:T
YFP47	C:T	A:G	A:A	C:C	A:A	G:A	G:G	A:A	A:A	C:C	A:A	T:G	G:G	A:A	G:G	G:A	T:T	C:T	T:T
YFP48	C:T	A:A	G:G	T:T	A:G	G:G	G:G	A:A	G:G	C:C	A:A	G:G	T:T	A:A	T:T	A:A	C:C	C:C	G:G
YFP49	C:T	G:G	A:A	T:C	A:A	G:A	G:G	NA	A:A	C:C	A:A	G:G	G:G	A:C	G:G	G:G	T:T	C:C	T:T

YFP50	C:T	A:G	A:A	T:C	A:G	G:A	G:C	C:A	G:G	C:C	A:G	T:T	G:T	A:C	T:G	A:A	T:T	C:T	T:T
YFP51	C:C	A:A	A:A	T:C	A:A	G:A	G:C	C:A	A:A	C:C	A:A	T:G	G:T	A:C	T:G	G:G	T:C	C:C	T:T
YFP52	C:C	G:G	G:G	T:C	A:G	NA	G:C	C:A	G:A	C:C	A:G	T:T	T:T	A:A	G:G	G:A	T:T	C:C	G:T
YFP53	C:C	A:A	A:A	T:C	A:G	A:A	G:C	C:A	G:G	C:C	A:A	T:T	G:T	A:C	G:G	G:G	C:C	C:C	G:T
YFP54	C:C	A:G	A:A	T:C	A:A	G:A	G:C	A:A	A:A	T:C	A:A	T:T	T:T	A:A	T:G	G:A	T:T	C:C	T:T
YFP55	T:T	A:G	A:A	T:C	A:A	G:A	C:C	C:A	G:G	T:T	A:A	T:T	T:T	C:C	T:T	A:A	T:T	T:T	T:T
YFP56	T:T	G:G	A:A	T:C	A:A	G:A	G:C	C:A	G:G	C:C	A:A	T:T	G:T	NA	G:G	A:A	T:T	C:C	T:T
YFP57	T:T	A:G	A:A	T:C	A:A	G:A	G:G	C:A	A:A	C:C	A:A	T:T	G:T	A:C	T:T	A:A	T:T	C:C	G:G
YFP58	T:T	A:G	A:G	T:T	A:G	A:A	G:G	C:A	G:A	T:T	A:A	T:G	T:T	A:A	G:G	G:A	T:T	T:T	G:G
